# The resistance mechanisms of proteasome inhibitor bortezomib

**DOI:** 10.1186/2050-7771-1-13

**Published:** 2013-03-01

**Authors:** Shuqing Lü, Jianmin Wang

**Affiliations:** 1 Department of Hematology, Changhai Hospital, Second Military Medical University, 168 Changhai Road, Shanghai 200433, China

**Keywords:** Drug resistance, Mechanism, Proteasome inhibitor, Bortezomib, PSMB5

## Abstract

The proteasome inhibitor, bortezomib, a boronic dipeptide which reversibly inhibit the chymotrypsin-like activity at the β5-subunit of proteasome (PSMB5), has marked efficacy against multiple myeloma and several non-Hodgkin’s lymphoma subtypes, and has a potential therapeutic role against other malignancy diseases. However, intrinsic and acquired resistance to bortezomib may limit its efficacy. In this article, we discuss recent advances in the molecular understanding of bortezomib resistance. Resistance mechanisms discussed include mutations of PSMB5 and the up-regulation of proteasome subunits, alterations of gene and protein expression in stress response, cell survival and antiapoptotic pathways, and multidrug resistance.

## Introduction

**Figure 1:**
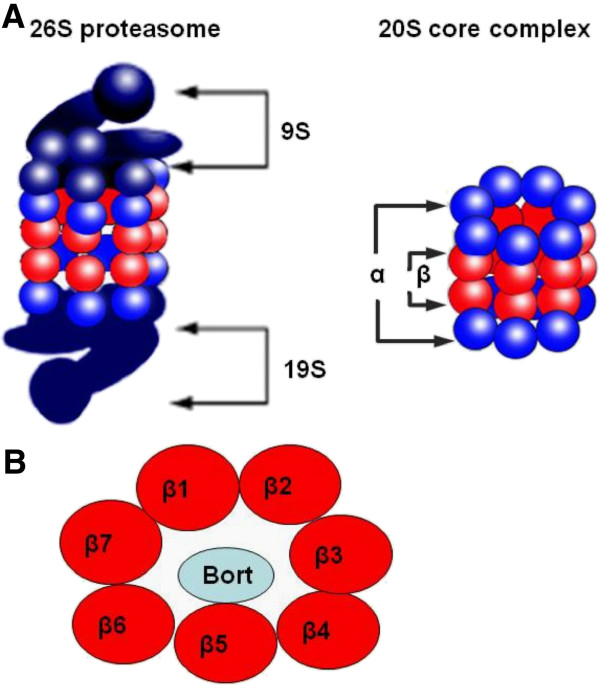
**The structure of 26 s proteasome. **The proteasome is a 26S enzyme complex that comprised of core 20S catalytic complex and 19S regulatory complex (**A**). The 20S proteasome core has chymotrypsin-like, trypsin-like, and peptidyl glutamyl-like activities that are associated with three distinct units: β5, β2, β1, respectively. Chymotrypsin-like activity at proteasome β5 subunit (PSMB5) is associated with the rate-limiting step of proteolysis. Proteasome inhibitor bortezomib (Bort) mainly inhibit the chymotrypsin-like activity at the PSMB5 (**B**).

The ubiquitin-proteasome pathway plays an essential role in the degradation of cellular proteins involved in a variety of cellular processes, including transcriptional regulation, cell cycle progression, proliferation, and apoptosis [1]. The proteasome has been well recognized as a valid target for anti-tumor therapy [2]. The proteasome is a 26S enzyme complex that comprised of 20S core complex and 19S regulatory complex (Figure 1A). Within the 20S core, proteins are degraded to small peptides. The 20S proteasome core has chymotrypsin-like, trypsin-like, and peptidyl glutamyl-like activities that are associated with three distinct units: β5, β2, β1, respectively. Chymotrypsin-like activity at proteasome β5 subunit (PSMB5) is associated with the rate-limiting step of proteolysis [3,4] (Figure 1B). It is conformationally flexible with active catalytic sites located on the inner surface of the cylinder where protein substrates bind [5-7]. Another form of the proteasome that is primarily expressed in cells of hematopoietic origin and cells exposed to inflammatory cytokines, known as the immunoproteasome (i20S), has the three catalytic activities represented by LMP7 (β5i), LMP2 (β1i), and MECL1 (β2i), which are more efficient in regulating antigen processing [8].

Inhibition of the proteasome results in perturbation of intracellular protein homeostasis by accumulation of the poly-ubiquitinated proteins, subsequently inducing cellular stress and apoptosis. Numerous proteasome inhibitors have been developed and described. Bortezomib (PS-341, Velcade), a boronic dipeptide which reversibly inhibit the chymotrypsin-like activity at the β5-subunit and to a lesser extent inhibit the trypsin-like activity at the β1-subunit, is the first proteasome inhibitor approved by FDA of the United States. Bortezomib induces apoptosis in a wide variety of cancer cell lines and other transformed cells, yet has relatively few toxic effects on normal cells [2,9,10]. Clinical studies have demonstrated the safety and promising efficacy of bortezomib as the single-agent or combined with other drugs against multiple myeloma (MM) [11,12], as well as in several non-Hodgkin’s lymphoma subtypes [13]. Bortezomib can also potentiate leukemia cell apoptosis and has a potential therapeutic role against leukemia, either alone or in combination with chemotherapy [14,15].

However, there are some patients who do not respond to therapy or respond briefly, then relapse. Here we will review the development of study in resistance mechanisms of bortezomib.

### Point mutation or over-expression of PSMB5 gene

To shed light on the mechanisms of acquired resistance to bortezomib after drug exposure, we established bortezomib-resistant lymphoblastic lymphoma/leukemia cells JurkatB, from the parental Jurkat cell line by repeated drug exposure and selection. First we confirmed G322A point mutation of PSMB5 gene confer bortezomib resistance to JurkatB cells. The inhibition rate of chymotrypsin-like activity in JurkatB cells was significantly decreased compared with parental Jurkat cells after bortezomib treatment. The retro-virally transduced Jurkat-mPSMB5 cells acquired a bortezomib-resistant phenotype due to the expression of G322A mutated PSMB5, reiterating the idea that the PSMB5 mutation is an important mechanism of bortezomib resistance [16]. Then JurkatB cells were selected with bortezomib at higher concentrations for extended periods of time, revealing novel PSMB5 mutants: C323 T mutant and G322A, C326 T conjoined mutant. The inhibitory effect of bortezomib on chymotrypsin-like activity was the weakest in JurkatB-G322A/C326T cells, and the strongest in JurkatB-G322A cells, with JurkatB-C323T cells falling in between. So the C323 T mutation and G322A, C326 T conjoined mutation of PSMB5 gene confer stronger bortezomib resistance than G322A mutation in JurkatB cells. The crystal structure in yeast illustrates that specific interactions are formed between the pyrazine-2-carboxyl side chain of bortezomib and residues of the PSMB5 specific pocket (S1 pocket). In the case of the chymotryptic-like active site, the carbonyl oxygen of bortezomib interact with PSMB5 Ala49 N and PSMB5 Ala50 N by a tight hydrogen bonding network. Computer modeling of the Ala49Thr-mutated PSMB5 protein resulting from the G322A mutation suggests that a conformation change occurs that may disrupt contacts between the chymotrypsin-like active site and bortezomib. The G322A, C323 T mutation and the G322A, C326 T conjoined mutation causes amino acid substitutions at position 108 and/or 109 of precursor PSMB5 protein (Ala108Thr and Ala109Val), which translates to Ala49Thr and/or Ala50Val of functional PSMB5 protein. So Ala49 and Ala50 of the PSMB5 protein are key positions relative to the inhibitory effect of bortezomib on chymotrypsin-like activity. Mutations in the PSMB5 gene that result in substitutions of these amino acid residues can confer varying bortezomib resistance [17].

Following our study, Oerlemans et al. also confirmed G322A PSMB5 mutation which resulted in Ala49Thr substitution in bortezomib resistant human monocytic/macrophage THP1/BTZ cells established by exposure to stepwise increasing concentrations of bortezomib [18]. Franke et al. confirmed the G322A, C323T PSMB5 mutations in bortezomib-resistant MM cell line 8226/BTZ and acute lymphoblastic leukemia cell line CEM/BTZ selected by bortezomib exposure [19]. Ri et al. also established two bortezomib-resistant MM cell lines KMS-11/BTZ and OPM-2/BTZ, and demonstrated these resistant MM cells have G322A mutation of PSMB5 gene. KMS-11 parental cells transfected to express mutated PSMB5 also showed reduced bortezomib induced apoptosis compared with those expressing wild-type PSMB5 or the parental cells. Expression of mutated PSMB5 was associated with the prevention of the accumulation of unfolded proteins and subsequent excessive endoplasmic reticulum (ER) stress which triggers apoptotic signals [20]. Otherwise, Franke et al. also identified G332T (Cys52Phe substitution) mutation in CEM/BTZ cells, G311T (Met45Ile substitution), A310G (Met45Val substitution) in THP1/BTZ cells and A247G (Thr21Ala substitution) mutation in 8226/BTZ cells [19]. Recently, de Wilt et al. confirmed acquired bortezomib resistance in non-small cell lung cancer (NSCLC) cell lines is associated with PSMB5 mutations resulting in Ala49Thr, Met45Val and Cys52Phe substitutions [21].

**Figure 2:**
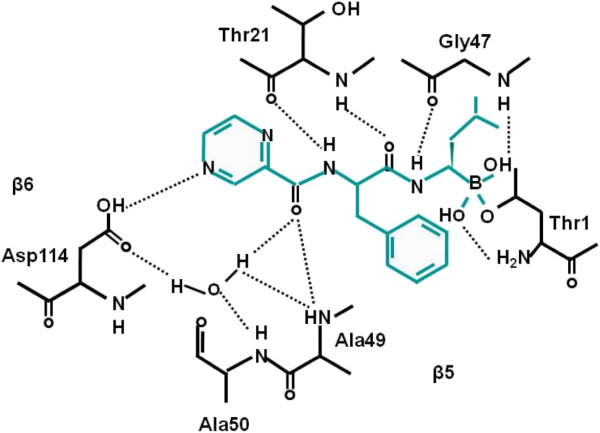
**Interaction model of bortezomib with active site of β5 subunit of proteasome.** Ala49, Ala50, Thr21 cited at the specific pocket of β5 subunit of proteasome (PSMB5). Ala49, Ala50, Thr21 participate in the tight hydrogen-bonding network, when bortezomib (in blue) interacts with the active site of PSMB5.

**Figure 3:**
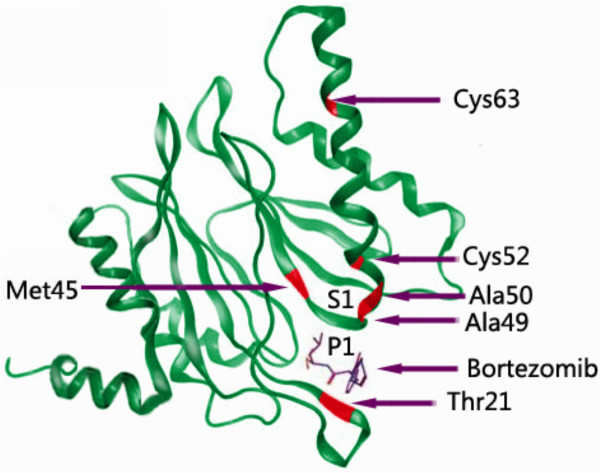
**The 3D protein-backbone structure of the β5 subunit of proteasome.** The 3D model of protein-backbone structure of the β5 subunit of proteasome (PSMB5) consults the yeast proteasomal crystal structure in complex with bortezomib [22]; the bovine proteasomal crystal structure [23]; models made by Franke et al [19],. de Wilt et al [21]. and Suzuki et al [24]. Mutation sites are depicted in red. P1, substrate side chain 1; S1, specificity binding pocket 1.

The vast majority of these mutated residues are around the S1 specificity pocket of the PSMB5. This S1 specificity pocket is mainly responsible for recognizing the peptide bond of the substrate. The Thr21Ala, Ala49Thr and Ala50Val substitutions in the bortezomib-binding pocket at the PSMB5 are directly involved in bortezomib binding to PSMB5 (Figure 2). Cys52Phe, Met45Ile substitutions in close proximity to the bortezomib-binding pocket in the PSMB5 are indirectly involved in bortezomib binding. Silico data suggest that all acquired mutations decrease the affinity of the PSMB5, particularly of the S1-binding pocket, to bortezomb (Figure 3, [22], [23]).

Otherwise, Suzuki et al. established clonal bortezomib resistant isolates of HT-29 adenocarcinoma cells adapted to continuous exposure of bortezomib. Two novel and distinct mutations in the PSMB5, Cys63Phe, located distal to the binding site in a helix critical for drug binding, and Arg24Cys, found in the propeptide region were found in all resistant clones. The portion containing Arg24 is required for proper subunit processing. It is possible that the altered recovery of proteasome activity following bortezomib exposure is a result of altered β5 processing [24]. The mutation Arg24Cys is a natural variant, with a frequency 5 times higher in patients with MM than in the general population reported by Wang et al [25]. Cys63 is housed in the same helix as Ala49/50, residues critical for bortezomib binding as described previously (Figure 3). The Cys63Phe mutation leads to a shift in the angle of the helix with respect to the active site, therefore, likely affects the binding of bortezomib to PSMB5. Of further note was the presence of a mutation in the propeptide portion of LMP7 (Phe50Ile) in 2 of 3 bortezomib resistant clonal isolates [24].

Interestingly, Kale et al. have observed Met45Val and Ala49Val mutations located within the S1 binding pocket of proteasome β-subunit in the irreversible proteasome inhibitor salinosporamide, producing marine actinobacterium Salinispora tropica, in which all seven β-subunits are identical. The mutant was markedly resistant to proteasome inhibitor bortezomib and salinosporamide than the wildtype Salinispora tropica. Met45Val played little role in resistance, whereas Ala49Val affected inhibitor resistance, substrate specificity, and prosequence cleavage. While the subunit topology and cellular function of the 20S proteasome in humans and actinobacteria are not identical, it is striking that equivalent mutations are utilized in both systems to achieve proteasome inhibitor resistance [26].

However, no mutations in PSMB5 have been detected in myeloma patients refractory to, or relapsed from, bortezomib treatment [27,28]. To further investigate the role of mutations of the PSMB5 in bortezomib resistance of clinical patients, a larger number of patients who have acquired bortezomib resistance during or after bortezomib treatment are required.

Our previous study also showed that the PSMB5 gene was over-expressed in some resistant JurkatB cells accompanied by increased chymotrypsin-like activity. We also found upregulated expression of the PSMB5 gene contributed to drug resistance in patient with multiple myeloma when treated with bortezomib-based regimen [29]. Similar discovery was reported by Oerlemans et al. in an acquired bortezomib resistant human monocytic/macrophage THP1/BTZ cell line [18] and by Balsas et al. in an acquired bortezomib resistant myeloma cell line [30]. Therefore, over-expression of the PSMB5 gene in response to drug stress, contributing to the increased chymotrypsinlike activity, is an important mechanism of acquired bortezomib resistance. Study of Yang YM et al. demonstrated that the PSMB5 downregulation by Ga12/13 inhibition enhances the anticancer effect of bortezomib, which may be of use to improve bortezomib therapy and reduce bortezomib resistance [31]. Former study by Kraus et al. showed that the proteasomal activity profile varies in primary leukemia cells, and that the pattern of proteasomal subunit activity influences the sensitivity of hematologic malignancies toward bortezomib [32]. de Wilt et al. also demonstrated that high basal levels of proteasome activity correlate with intrinsic bortezomib resistance in various NSCLC cell lines, and acquired bortezomib resistance in NSCLC, is associated with proteasome subunit overexpression [21]. Proteasome activity and levels of both the constitutive and immunoproteasome were increased in bortezomib resistant HT-29 adenocarcinoma cells, which correlated to an increase in subunit gene expression [24].

The results of these studies are important for the design of novel proteasome inhibitors without cross-resistance with bortezomib.

Study of de Wilt et al. showed there was obvious cross-resistance in some of the bortezomib resistant NSCLC cell lines harboring PSMB5 mutations to other proteasome inhibitors specifically targeting β-subunits (including MG132 which targets all β subunits of the proteasome, irreversible proteasome inhibitors, such as carfilzomib and an derivative ONX 0912). Carfilzomib, an irreversible tetrapeptide epoxyketone proteasome inhibitor, exhibits more specificity for the chymotrypsin-like activity at the β5 and immuno-β5-subunit than other proteasome inhibitor, and appears to have a more favorable toxicity profile. ONX 0912, a tripeptide epoxyketone, is an orally available analogue of carfilzomib. But there was no obvious cross-resistance in these bortezomib resistant NSCLC cells to the novel α7-subunit-specific proteasome inhibitor (5-amino-8-hydroxyquinoline, 5-HAQ) [21]. This is similar to study of Franke et al. Bortezomib resistant (170-fold) acute lymphoblastic leukemia CEM/BTZ cells gained a cross-resistance to carfilzomib (39-fold). THP1 myeloid sublines with acquired resistance to bortezomib (54–235 fold) caused by mutations in the PSMB5 gene displayed less pronounced cross-resistance to carfilzomib (9–32 fold) [19]. But, irreversible proteasome inhibitor carfilzomib showed only a 3–4 fold decrease in cytotoxic potential in the bortezomib resistant HT-29 adenocarcinoma cells, another irreversible proteasome inhibitor, LLL-Bor, was also equivalently cytotoxic to both resistant and parental cells, while the bortezomib resistant cells remained refractory to the reversible inhibitor MG132 [24]. These results may have important clinical implications. The second-generation proteasome inhibitors directed against α subunits can overcome this bortezomib resistance in cells harboring PSMB5 mutations, hence offering a potential future treatment modality. Resistance to bortezomib, can be partially overcome with irreversible inhibitors, suggesting prolonged proteasome inhibition induces a more potent anti-tumor response. An open-label, single-arm, phase 2 study showed single-agent carfilzomib had activity in patients with relapsed and/or refractory MM who had received prior treatment with bortezomib [33]. Moreover, some studies showed ubiquitination pathway enzyme inhibitors achieve the same effect as protesome inhibitor, the disregulation of cellular protein destruction, with an alternative target. Thus, inhibition of the ubiquitination pathway enzyme is a novel strategy to overcome bortezomib resistance [34-36].

### Alterations of gene and protein expression in stress response,cell survival and antiapoptotic pathways

Bortezomib, inhibiting the chymotrypsin-like activity at the PSMB5, blocked the degradation of the poly-ubiquitinated proteins such as IkB-.alpha;, a negative regulator of the nuclear factor (NF)-κB pathway, and unfolded or oxidatively modified proteins followed by endoplasmic reticulum (ER) stress and effects on the tumor microenvironment associated apoptosis [4]. Thus, the upregulation of gene and protein expression in stress response and cell survival, antiapoptotic pathways can confer tumor cells bortezomib resistance.

Shringarpure et al. demonstrated that heat shock proteins (HSPs: HSP70, HSP27, HSP90) and other proteins with chaperone-like functions had higher expression levels in primary bortezomib resistant diffuse large B-cell lymphoma cell line SUDHL-4 cells than in bortezomib-sensitive SUDHL-6 cells. Blocking Hsp27 using an antisense strategy restores the apoptotic response to bortezomib in DHL4 cells; conversely, ectopic expression of wild-type Hsp27 renders bortezomib-sensitive DHL6 cells resistant to bortezomib. These data confirm that higher expression of HSPs and other chaperones is associated with resistance to bortezomib. The increased levels of chaperones including Hsp27 also prevent the activation of the unfolded protein response in ER and subsequent apoptosis [37-39]. Increased expression of HSP27 was also noted in bortezomib resistant HT-29 adenocarcinoma cells [24].

Bortezomib induced a significant upregulation of ATF 3, ATF 4, ATF 5, c-Jun and Jun D proto-oncogene in SUDHL-6 cells. Activation of JNK-1 in response to bortezomib was confirmed by an increase in phospho-JNK1 as well as increased phosphorylation of JNK substrates, c-Jun and ATF-2 in sensitive SUDHL-6 cells. However, no similar activation of JNK-1 in response to bortezomib in resistant SUDHL-4 cells was observed. The expression of transcription factor 4 (TCF-4) was 15-fold higher in resistant SUDHL-4 cells compared with sensitive SUDHL-6 cell. TCF-4 is a central player in the Wnt signalling pathway that has been implicated in cancer development, differentiation, and drug resistance. Potential downstream target genes of the TCF-4/β-catenin complex, cyclin D1 and c-myc, were also upregulated in SUDHL-4 cells. So the differential gene expression profiles of SUDHL-4 and SUDHL-6 cells have confirmed the activation of pathways mediating bortezomib induced apoptosis in sensitive SUDHL-6 cells, but not in resistant SUDHL-4 cells. These studies will provide valuable insights into the mechanisms of drug resistance to bortezomib, and identify molecular targets to overcome bortezomib resistance in haematological malignancies. β-catenin, the key protein in canonical Wnt pathway, degrades via ubiquitin-proteasome pathway. Study of Zhou et al. found that myeloma cell lines with higher β-catenin level are less sensitive to bortezomib, and combination treatment of low dose 2-methoxyestradiol, arsenic trioxide and bortezomib can reduce β-catenin accumulation and enhance the sensitivity to bortezomib [36,39,40].

Interleukin-6 and insulin-like growth factor (IGF-1) in the microenvironment can also maintain cell growth, and confer resistance to bortezomib by activation of NF-ΚB through Raf/MEKK1 and PI3-K/Akt pathways [4,41]. Study of Wang et al. and Zang et al. showed bone marrow stromal cells (BMSCs) regulate the drug sensitivity of myeloma cells through the inhibited expression of miRNA and IL-6 plays a pivotal role in the occurrence of drug resistance [42,43]. IGF-1 produced by plasma cells, as well as by the marrow microenvironment, is a critical mediator of a number of downstream effects that contribute to multiple myeloma pathobiology. The IGF-1 receptor has been found to be over-expressed in myeloma and this aberrant expression, as well as higher IGF-1 levels, have been related to disease progression, severity, and prognosis [44-46]. Studies of Kuhn et al. revealed evidence that increased IGF-1 signaling through enhanced IGF-1 secretion and IGF-1R activation was associated with the phenotype of resistance in bortezomib-resistant cell lines selected from RPMI 8226, OPM-2, ANBL-6, and KAS-6/1 myeloma cell lines by bortezomib exposure and no PSMB5 mutations were found in these bortezomib-resistant cell lines. Additionally, gene expression profiling confirmed that genes acutely activated by IGF-1 stimulation were chronically expressed in bortezomib-resistance cell lines. Furthermore, blockade of downstream targets such as PI3K and mTOR could, to some extent, overcome this resistance. Pharmacologic or genetic suppression of IGF-1R also sensitized cell lines and patient samples to bortezomib therapy. Finally, the IGF-1R inhibitor OSI-906 synergized with bortezomib to enhance myeloma cell death and overcame bortezomib-resistance in vivo. Thus, these data strongly implicate that signaling through the IGF-1/IGF-1R axis contributes to acquired bortezomib resistance, and provide a rationale for combining bortezomib with IGF-1R inhibitors likely to overcome or possibly prevent the emergence of bortezomib resistance [47].

Study of Que et al. showed c-Met, a receptor tyrosine kinase, is over-expressed in human myeloma cell lines and promotes the survival and drug resistance of myeloma cells. This study confirmed knockdown of c-Met enhances sensitivity to bortezomib in human multiple myeloma U266 cells via inhibiting Akt/mTOR activity [48]. Kim et al. generated bortezomib-resistant mantle cell lymphoma cell lines and found increased phosphorylation of Akt and mTOR. Dual inhibition of PI3K and mTOR with BEZ235 could overcome acquired resistance to bortezomib in mantle cell lymphoma cells and suppress the activated Akt/mTOR pathway [49].

Moreover, microarray analysis revealed that the mRNA levels of Rad (Ras associated with diabetes) were higher in the bortezomib-resistant Jurkat-R cells than in the parental control cells. Rad knockdown overcame bortezomib resistance and induced mitochondrial apoptosis via Noxa/Bcl-2 modulation. Rad decreased cell death in response to bortezomib. Leukemia and lymphoma cell lines (K-562, Raji, IM-9 and Jurkat-R) with elevated Rad expression levels showed higher degrees of bortezomib resistance versus those (Sup-B15, JVM-2, U266 and Jurkat) with low Rad expression levels [50]. Study of Gu et al. demonstrated that myeloma differentiation status is associated with myeloma sensitivity to bortezomib and that induction of differentiation, increasing the proteasome workload in myeloma cells by increasing immunoglobulin secretion, while reducing proteasome capacity by decreasing proteasome activity, can overcome myeloma resistance to bortezomib [51]. Jung et al. reported that stem-like cells in mantle cell lymphoma, termed mantle cell lymphoma-initiating cells, enriched in the population that lack prototypic B-cell marker CD19. These cells were able to self-renew upon serial transplantation and are highly tumorigenic. Importantly, these stem-like cells are resistant to bortezomib, as well as chemotherapeutic regimens containing bortezomib, despite constitutive NF-κB expression [52]. Molecules targeting these spots prospectively overcome bortezomib resistance and possibly prevent the emergence of bortezomib-refractory disease in the clinic.

### Multi-drug resistance (MDR)

In our previous study, no cross-resistance to anthracycline, alkaloid, or topoisomerase inhibitor drugs was displayed in bortezomib-resistant JurkatB cell lines. The result of a flow cytometric assay utilizing daunorubicin (DNR) suggested that the bortezomib-resistant JurkatB cells did not gain drug efflux function. Meanwhile, expression of P-glycoprotein (P-gp) was not detected in all JurkatB lines by western blot. No significant differences of mRNA expression levels of MDR1, lung resistance-related protein (LRP), multi-drug resistance-associated protein (MRP) genes were shown between JurkatB and Jurkat cells. We thus concluded that MDR is unlikely to account for the aquired bortezomib resistance in JurkatB lines, which is similar to the results observed in the primary bortezomib resistant SUDHL-4 B-lymphoma cells [29,37]. De Wilt et al. also found no cross-resistance to a prototypical P-gp substrate (i.e. DNR) and differences in functional P-gp activity between parental and bortezomib resistant cells, suggesting that enhanced drug efflux via MDR transporters does not contribute to bortezomib resistance in NSCLC cells [21].

Our previous study showed a significant cytotoxicity of bortezomib on a P-gp positive leukemia line K562/A02 cells, but the effect on K562/A02 cells was significantly decreased compared to that on K562 cells. The resistance-fold of K562/A02 cells to bortezomib compared with K562 cells is much lower than to anthracycline. The resistance-fold of K562/A02 to adriamycin and DNR were 139 and 67 compared with K562 cells, while the resistance-fold of K562/A02 to bortezomib was only 3 compared with K562 cells. These results showed only a slight cross-resistance of K562/A02 to bortezomib [53]. Lymphoid CEM/VLB cells with P-gp overexpression exhibited substantial resistance to carfilzomib (114-fold), whereas less resistance to bortezomib (4.5-fold) was observed [54]. Minderman et al. showed that among P-gp, breast cancer resistance protein and LRP, only P-gp conferred resistance to bortezomib, and the resistance was only two-fold [55]. Rumpold et al. showed that knockdown of MDR1 resensitizes leukemic cells to bortezomib, suggesting bortezomib is also a P-gp substrate, which might be relevant for drug-resistance in cancer [56].

To sum up, though drug extrusion via the multidrug efflux transporter P-gp has been shown to mediate a low level of bortezomib resistance, MDR may be not the overwhelming mechanism of bortezomib resistance.

## Conclusions

**Figure 4:**
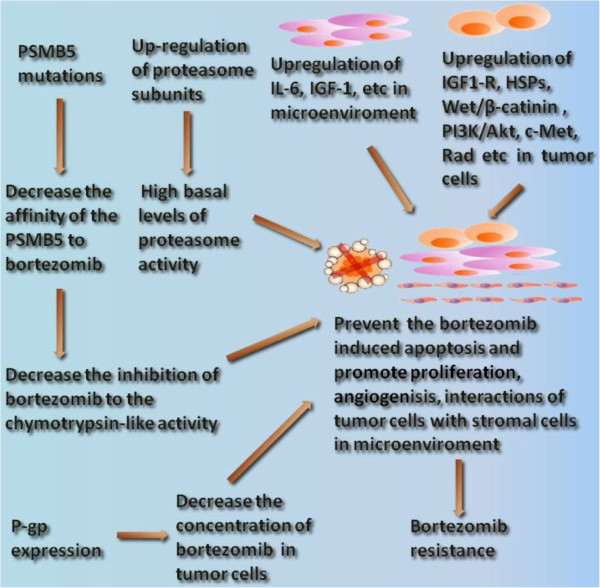
**The summary of possible bortezomin resistance mechanisms. **The mechanisms of proteasome inhibitor bortezomib resistance mainly focused on the modifications of the mechanisms of its action. Mutations at key points of β5 subunit of proteasome (PSMB5) decrease the affinity of the PSMB5 to bortezomb, which result in the decreased inhibition of the chymotrypsin-like activity, up-regulation of proteasome subunits contributing to the high basal levels of proteasome activity, alterations of gene and protein expression in stress response and cell survival, antiapoptotic pathways are important mechanisms of bortezomib resistance. Efflux transporter P-gp was also shown to mediate a certain degree bortezomib resistance.

The mechanism of proteasome inhibitor bortezomib resistance mainly focused on the modifications of the mechanisms of its action. The possible bortezomib resistance mechanism can be summarized as Figure 4.

Bortezomib mainly reversibly inhibit the chymotrypsin-like activity at the PSMB5 and to a lesser extent inhibit the trypsin-like activity at the β1 subunit. The mutation and over-expression of β subunits is a hotspot in the study of bortezomib resistance. Our previous study and other teams’ studies demonstrated point mutations of PSMB5 is an important mechanism of bortezomib resistance. The majority of the mutations reside around the S1 specificity pocket of the PSMB5. The Thr21Ala, Ala49Thr and Ala50Val substitutions in the bortezomib-binding pocket in the PSMB5 are directly involved in bortezomib binding to PSMB5. Cys52Phe, Met45Ile substitutions in close proximity to the bortezomib-binding pocket in the PSMB5 are indirectly involved bortezomib binding. The Cys63Phe mutation housed in the same helix as Ala49/50 leads to a shift in the angle of the helix with respect to the active site, therefore, likely affects the binding of bortezomib to PSMB5. Silico data suggest that all acquired mutations decrease the affinity of the PSMB5 to bortezomb, resulting in the decreased inhibition of the chymotrypsin-like activity, then displayed bortezomib resistance [16-21]. Furthermore, studies have demonstrated that overexpression of the PSMB5 gene and the up-regulation of proteasome subunits contributing to the high basal levels of proteasome activity correlate with acquired and intrinsic bortezomib resistance.

The results of these studies are important for the design of novel proteasome inhibitors without cross-resistance with bortezomib. Proteasome inhibitors specific for other subunits, such as the novel α7-subunit-specific proteasome inhibitor 5-HAQ, have been reported to be susceptible to tumor cells with β5 subunit up-regulation or mutation. Moreover, resistance to bortezomib can be partially overcome with irreversible inhibitor carfilzomib. In addition, inhibition of the ubiquitination pathway enzyme is also a novel strategy to overcome bortezomib resistance.

The anti-tumor effects of bortezomib are a result of cell cycle arrest and apoptosis resulting from blocking the degradation of the poly-ubiquitinated proteins after proteasome inhibition. The underlying mechanisms include NF-κB inhibition, upregulation of various apoptotic pathways, downregulation of antiapoptotic pathways and effects on the tumor microenvironment. So, alterations of gene and protein expression in stress response and cell survival, antiapoptotic pathways are also important mechanisms of bortezomib. For example, upregulation of HSPs, IL-6, IGF-1/IGF-1R, c-Met, Rad, β-catenin/Wnt and Akt/mTOR pathways were confirmed to be associated with bortezomib resistance. Molecules targeting these spots prospectively overcome bortezomib resistance and possibly prevent the emergence of bortezomib-refractory disease in the clinic.

At last, MDR have been confirmed not to be the overwhelming mechanisms of bortezomib resistance in many studies. But in some other studies drug extrusion via the multidrug efflux transporter P-gp has been shown to mediate a low level of bortezomib resistance.

## Abbreviations

PSMB5: β5-subunit of proteasome; MM: Multiple myeloma; NF-κB: Nuclear factor-κB; P-gp: P-glycoprotein; MDR: Multidrug resistance; HSPs: Heat shock proteins:HSPs; IL-6: Interleukin-6; IGF-1: Insulin-like growth factor 1; IGF-1R: Insulin-like growth factor 1 receptor; 5-HAQ: 5-amino-8-hydroxyquinoline; NSCLC: Non-small cell lung cancer; ER: Endoplasmic reticulum, DNR: daunorubicin.

## Competing interests

The authors declare that they have no competing interests.

## Authors’ contributions

SL wrote the paper, JW revised the paper. Both authors read and approved the final manuscript.
